# Determinants of Hypertension Among Transport Workers in Ibadan, Nigeria: A Structural Equation Modeling Approach

**DOI:** 10.7759/cureus.96458

**Published:** 2025-11-09

**Authors:** Olajide Akinpeloye, Akpa Onoja, Abdulazeez Alabi

**Affiliations:** 1 Department of Mathematics, Georgia State University, Atlanta, USA; 2 Department of Epidemiology and Medical Statistics, University of Ibadan, Ibadan, NGA; 3 Division of Epidemiology, Biostatistics, and Environmental Health, University of Memphis, Memphis, USA

**Keywords:** cardiovascular disease, hypertension, lifestyle factors, structural equation modelling (sem), transport workers

## Abstract

Introduction

Hypertension is one of the most potent risk factors for cardiovascular disease (CVD). Transport workers are frequently exposed to multiple health risks, and undiagnosed hypertension among commercial drivers may result in severe road accidents.

Objective

The study aimed at assessing the multidimensional relationship between risk factors for hypertension among transport workers in Nigeria.

Methodology

A cross-sectional study was conducted among 682 transport workers (≥18 years) recruited from motor parks across the Ibadan metropolis. Data were extracted from the Community-Based Investigation of the Risk Factors for Cardiovascular Diseases (COMBAT-CVDs) study. The WHO STEPwise instrument was used to collect information on sociodemographic, lifestyle, and body fat distribution characteristics. Blood pressure and anthropometric measurements were recorded. Hypertension was defined as systolic blood pressure ≥140 mmHg and/or diastolic blood pressure ≥90 mmHg. Data were analyzed using descriptive statistics, chi-square test (α = 5%), and structural equation modeling (SEM).

Results

The mean age of respondents was 42.4 ± 7.9 years. The overall prevalence of hypertension was 33.3% (227, 95% confidence interval (CI): 29.8%-36.8%). The highest prevalence was observed among participants aged 45-64 years (123, 45.2%), followed by those aged ≥65 years (16, 45.7%). Married participants had a higher prevalence of hypertension (198, 37.4%) compared with unmarried participants (29, 19.0%). Obesity, based on body mass index (BMI) classification, was present in 324 (47.5%) participants; individuals with abnormal BMI exhibited a higher prevalence of hypertension (129, 39.8%) than those with normal BMI (98, 27.4%). A history of smoking was reported in 72 (41.1%) hypertensive participants, while 115 (40.5%) reported alcohol consumption. Poor sleep quality was associated with hypertension in 93 (30.5%) participants, compared with 134 (35.5%) among those with good sleep quality. The SEM demonstrated a good overall fit (root mean square error of approximation (RMSEA) = 0.04, Goodness of Fit Index (GFI) = 0.90, Tucker-Lewis Index (TLI) = 0.90, and Comparative Fit Index (CFI) = 0.91). Among the latent constructs, age exerted the strongest total effect on hypertension (β = 0.214), followed by body-fat distribution (0.114), lifestyle (-0.147), sociodemographic characteristics (0.069), and physical inactivity (0.032).

Conclusions

Hypertension was common among transport workers and was influenced by sociodemographic, lifestyle, and body fat distribution factors. Age and central obesity were the strongest predictors, while modifiable lifestyle behaviors such as smoking, alcohol intake, and physical inactivity also contributed significantly. These findings highlight the need for targeted interventions and workplace health programs to reduce hypertension risk in this group.

## Introduction

Hypertension, often referred to as the *silent killer*, remains one of the most critical global public health challenges [1]. It is a leading risk factor for cardiovascular disease (CVD) and the most common cause of stroke. Complications of elevated blood pressure include heart failure, peripheral vascular disease, renal impairment, and retinal damage [2].

According to the World Health Organization (WHO) 2024 Hypertension Fact Sheet, an estimated 1.4 billion adults aged 30-79 years, about one-third of the global population in this age group, live with hypertension, and two-thirds of them reside in low- and middle-income countries [2]. Alarmingly, approximately 600 million adults with hypertension (44%) are unaware of their condition, and only about 23% have it adequately controlled. Hypertension remains a major cause of premature death worldwide, prompting a global target to reduce the prevalence of uncontrolled hypertension by 25% between 2010 and 2025. Despite these global efforts, prevalence continues to rise, driven by aging, obesity, physical inactivity, and unhealthy dietary patterns [2].

In Nigeria, hypertension contributes substantially to the nation’s low life expectancy, currently estimated at 62.6 years, ranking the country 167th globally [3]. As a major risk factor for CVD, hypertension accounts for a large proportion of CVD-related mortality [4]. Of the 29% of all deaths in Nigeria attributed to noncommunicable diseases (NCDs), CVDs contribute approximately 11% [5]. Therefore, identifying high-risk individuals and the factors influencing hypertension prevalence remains a public health priority. Transport workers are considered a particularly high-risk group due to unhealthy lifestyle behaviors such as smoking while driving, alcohol consumption before work, long working hours, and high occupational stress [6]. Hypertension has been reported to be common among commercial transport workers, with a prevalence of about 27.7%. This high rate has been linked to population growth, aging, and behavioral risk factors such as smoking, physical inactivity, excessive alcohol consumption, stress, and poor diet [4].

The transportation sector plays an important role in national development and is often considered a proxy for measuring urbanization and industrialization. Commercial driving has been reported as the leading cause of occupation-related mortality, accounting for nearly half of all occupational injury deaths in the United States [7]. Because of the nature of their work, commercial drivers often face high levels of stress, consume unhealthy diets, smoke while driving, and may use alcohol before or during trips, while rarely making time for medical check-ups [6]. Even among those receiving treatment for hypertension, poor medication adherence is common. As a result, many drivers continue their routine activities while at risk, contributing to frequent road accidents and a significant number of fatalities among both drivers and passengers [8].

However, studies exist on hypertension among commercial drivers in other countries such as Nigeria [9] India [10], Iran [11], Korea [12] with high effect record of hypertension among the identified group and the prevalence are higher than the reported prevalence of hypertension among commercial taxi drivers across Africa [13] and several developing countries [14] with ranges from 9.0% to 46%. Increasing trends in the occurrence of hypertension risk factors and their effects among professional drivers have been reported among transport workers in studies [15]. Findings from a study of app-based cab drivers in India indicated that nearly one in every two drivers reported at least one major NCD risk behavior (including tobacco use, alcohol consumption, sedentary lifestyle, and unhealthy diet), with 78 % physically inactive and 22 % using tobacco [2]. In Nigeria, a study reported that 57.6% of tanker drivers in Lagos consumed alcohol, a prevalence similar to the 54.2% observed among drivers in Benin City [16]. The prevalence of hypertension among commercial drivers in Jabi, Abuja is 9% prevalence and the national figure is 5%-7% [9].

Understanding the distribution of risk factors and their associated burden among commercial drivers is essential for effective preventive strategies in the fight against hypertension. This will provide evidence-based data for deciding what type of preventative action to design to tackle the threat. Genetic and demographic factors include age, gender, ethnicity, and educational level, and are unmodifiable, while behavioral factors such as physical inactivity, unhealthy dietary choices are often modifiable [17]. Genetic factors range from age, gender, body physique, with family history, and lifestyle factors include uncontrolled drinking, smoking, poor eating habits, and reduced physical activity [18]. Most of the factors contributing to the high burden of hypertension among commercial taxi drivers are modifiable with adequate intervention strategies needed to safeguard the health [15]. Professional driving puts drivers under stressful conditions, such as a sedentary lifestyle, unhealthy diet plan, irregular work schedules, and external stressors [19].

Structural equation modeling (SEM) is a statistical framework that allows researchers to estimate and test multiple interrelated regression equations within a single analysis. Unlike conventional regression, SEM incorporates both observed variables (e.g., age or gender) and latent constructs, which are unobservable factors inferred from measured indicators [20]. This approach is particularly valuable when relationships among risk factors are complex and involve both direct and indirect pathways. Unlike traditional regression models that treat each predictor as acting independently, SEM enables the simultaneous assessment of how several risk factors may influence one another and, together, an outcome such as elevated blood pressure [21]. This approach is particularly advantageous in public health research, where risk factors such as age, lifestyle, and metabolic indicators are often interrelated and influence disease outcomes through both direct and indirect pathways.

Previous research in Iran demonstrated the value of applying SEM to hypertension, showing how the technique can capture both direct and indirect effects of behavioral and demographic risk factors on the condition [22]. Building on this evidence, the present study applies SEM to examine the multidimensional links between lifestyle, demographic, and occupational risk factors and hypertension among commercial transport workers in Ibadan, Nigeria.

The conceptual framework guiding this study is described below to summarize the hypothesized pathways between sociodemographic, lifestyle, and body fat distribution factors and hypertension.

## Materials and methods

Conceptual framework

**Figure 1:**
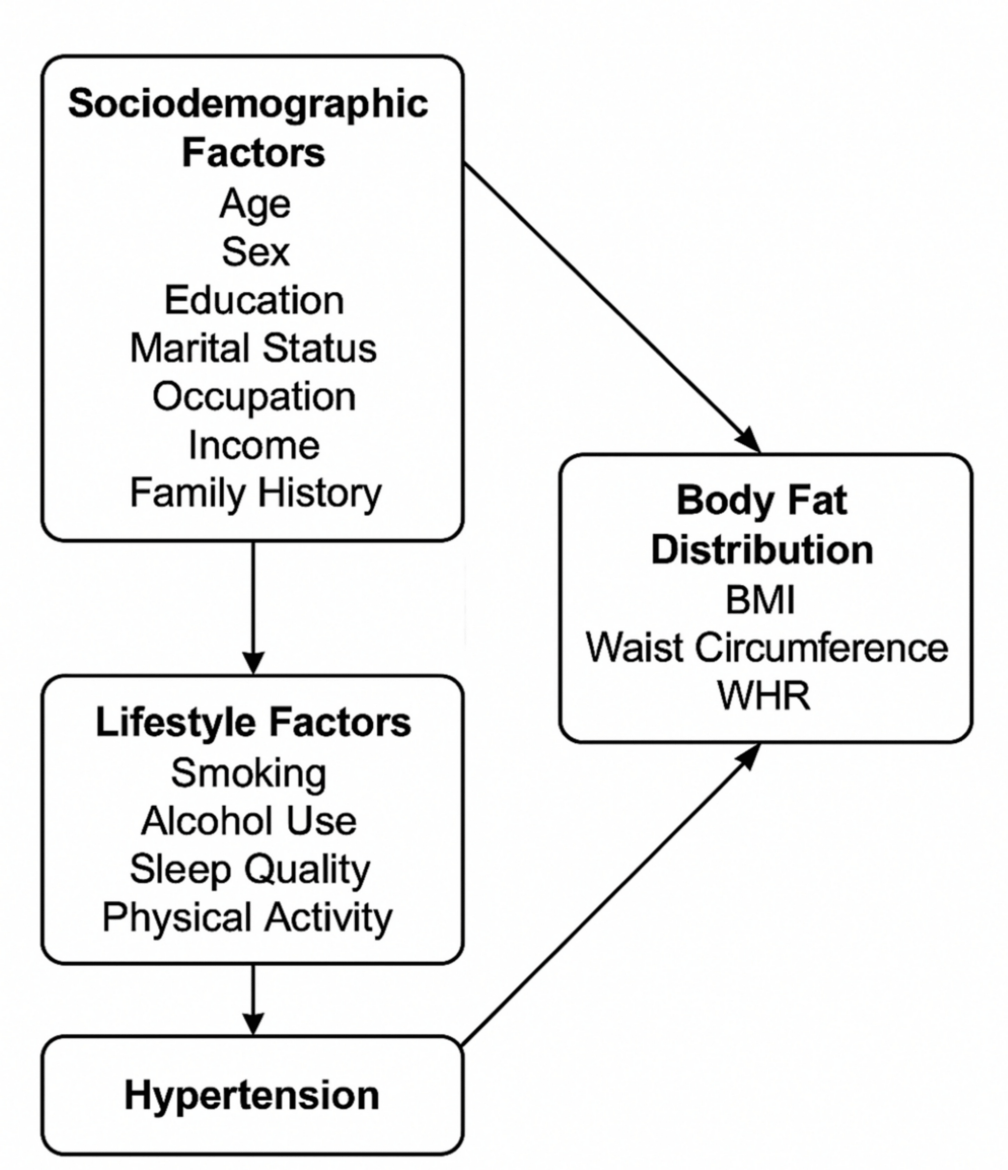
Conceptual framework of the hypothesized relationships among sociodemographic, lifestyle, and body fat distribution factors influencing hypertension among transport workers in Ibadan, Nigeria. Image credit: Created by Olajide Akinpeloye using Microsoft PowerPoint to design the conceptual framework. WHR, waist-to-hip ratio

The conceptual framework for this study describes how different factors are thought to interact in shaping the risk of hypertension among transport workers. Sociodemographic characteristics such as age, sex, education, marital status, occupation, income, and family history may have a direct influence on blood pressure or may act indirectly through their effects on lifestyle. Lifestyle factors such as smoking, alcohol use, sleep quality, and physical activity are expected to mediate these influences, while measures of body fat distribution, including BMI, waist circumference (WC), and waist-to-hip ratio (WHR), represent the physiological link between behavioral and demographic factors and elevated blood pressure. SEM was applied to capture both the direct and indirect effects among these interconnected variables (Figure 1).

Sociodemographic factors may affect hypertension directly or indirectly through their impact on lifestyle and body composition, while lifestyle behaviors and body fat measures together contribute to variations in blood pressure levels.

Study area and study population

Ibadan is one of the major urban centers in Nigeria, ranking as the third most populous city, with Lagos and Kano being the first and second, respectively. The city of Ibadan is located approximately at longitude 3°5ʹ East of the Greenwich Meridian and latitude 7°2ʹ North of the Equator at some 145 km east of Lagos. The city of Ibadan is made up of 11 local government areas (LGAs) [with an estimated population of 2,550,593 [23], 5 of which constitute the urban (Metropolitan) local governments, while the remaining six form the peri-urban or rural local governments. The study covered randomly selected LGAs from the Ibadan Metropolis and the Pre-urban or rural LGAs. Ibadan is home to a number of institutions: the commercial transportation system is overseen by the National Union of Road Transport Workers (NURTW). Study participants were selected from transport parks regulated by the NURTW. These parks host a variety of activities because they house commercial drivers, members of the transport union, and roadside touts. Traders in the parks included food vendors, hawkers of various commodities, sellers of cigarettes, alcoholic beverages, and alcohol-based herbal mixtures, as well as individuals dealing in illegal goods and contraband; these groups were also recruited for the study.

Data source and sample size

Data for this study were extracted from the Community-Based Investigation of the Risk Factors for Cardiovascular Diseases (COMBAT-CVDs) survey, a large community-based investigation of cardiovascular risk factors conducted in Ibadan and its suburbs [24]. Eligible participants for the original COMBAT-CVDs survey were selected through a multistage sampling technique. Communities in Ibadan and surrounding areas where respondents were accessible during working hours were purposively selected from two semi-urban and three urban LGAs. Within each selected community, a door-to-door approach was used to recruit consenting adults until the proportionally allocated sample size was achieved.

For the present study, one major motor park was selected from each of the five participating LGAs, and all consenting transport workers within those parks were included. From the broader COMBAT-CVDs dataset, a total of 682 transport workers aged 18 years and above who met the inclusion criteria were extracted for analysis. These participants included drivers, conductors, and traders operating within motor parks regulated by the NURTW across the Ibadan metropolis. The COMBAT-CVDs survey was conducted between February and May 2021, and data were collected during this period (AD 13/479/2029).

Data measurement

High blood pressure was defined as systolic or diastolic blood pressure readings ≥140/90 mmHg and/or self-reported use of antihypertensive medication within the previous two weeks, in accordance with the WHO/International Society of Hypertension guidelines [25]. BMI was calculated by dividing weight (kg) by the square of height (m²) and categorized using WHO standards as underweight (BMI <18.5), normal (18.5-24.9), overweight (25.0-29.9), and obese (≥30.0) [26]. WHR was obtained by dividing WC (cm) by hip circumference (cm), with central obesity defined as WHR >0.85 for women and >0.90 for men [27].

Data analysis

All statistical analyses were conducted using SPSS software (version 25.0; IBM Corp., Armonk, NY). Descriptive statistics and chi-square tests were carried out in SPSS, while SEM was performed in AMOS (version 22.0) applying the maximum likelihood estimation approach. Statistical significance was set at a *P*-value <0.05. The outcome variable for this study was hypertension, while the independent variables were socio-demographic characteristics such as age, marital status, gender, educational level, and family history of hypertension. Body fat distribution variables (BMI, WHR, and WC) and lifestyle variables (smoking status, alcohol intake, frequent fruit consumption/diet, physical activity, sleep quality, and stress). The independent variables were also considered as the risk factors for hypertension among the participants. Descriptive statistics such as mean, frequency, and proportion were used to summarize the data, and inferential statistics such as the chi-square test were used to test for the association between the categorical variables.

SEM was employed to examine the influence of risk factors on hypertension. The model pathways were specified based on theoretical foundations, prior studies, and expert input. SEM integrates measurement models, which describe how observed indicators represent latent constructs, and structural models, which capture the relationships among variables. This approach allows simultaneous estimation of direct and indirect effects while testing hypothesized associations [20].

SEM aims to assess whether the hypothesized covariance structure is consistent with observed data. In this study, the conceptual model incorporated sociodemographic factors, lifestyle behaviors, body fat distribution, and blood pressure. The modeling procedure involved specifying the conceptual model, verifying model identification, and evaluating goodness of fit. Model adequacy was assessed using common indices such as the Comparative Fit Index (CFI), Tucker-Lewis Index (TLI), and the root mean square error of approximation (RMSEA), which are widely recommended for SEM evaluation [28].

Missing data were handled using multiple imputation techniques to minimize bias and preserve statistical power. The imputation model accounted for the pattern and mechanism of missingness and included relevant confounders, consistent with recommended best practices in epidemiological analysis. 

Ethical considerations

Ethical approval for the original COMBAT-CVDs survey was obtained from the Oyo State Ministry of Health Research Ethics Committee (Reference No: AD13/479/2029A). This analysis used de-identified secondary data; therefore, additional participant consent was not required.

## Results

Of the 682 transport workers extracted for the study, 543 (79.3%) were males and 139 (20.7%) were females. The mean age of the respondents was 42.4 years; 467 (68.5%) were transport drivers, conductors, and park managers (NURTW workers), and 215 (31.5%) were traders around the motor parks covered. Most of the respondents (529, 77.6%) were married. Participants were predominantly Yoruba (661, 96.9%), while 21 (3.1%) were from other tribes. No formal, primary, secondary, and tertiary education was found to be 4.0%, 27.1%, 56.5%, and 12.5% respectively. On average, 176 (25.8%) earned less than 24,900 naira in a month, while 506 (74.2%) earned more than 24,900 naira in a month. Almost all (641, 94.0%) of the participants can trace hypertension to at least one family member. The overall prevalence of hypertension among transport workers was estimated to be 227 (33.3%) using a cutoff of 140/90 mmHg (Table 1).

**Table 1:** Sociodemographic characteristics of study participants (N = 682). NGN, Nigerian Naira

Variable	Category	*n* (%)
Age (years)	<25	70 (10.3)
	24-34	121 (17.7)
	35-44	184 (27.0)
	45-64	272 (39.9)
	≥65	35 (5.1)
Gender	Male	543 (79.3)
	Female	139 (20.4)
Marital status	Married	529 (77.6)
	Unmarried	153 (22.4)
Occupation	Transporter	467 (68.5)
	Non-transporter	215 (31.5)
Ethnicity	Yoruba	661 (96.9)
	Others	21 (3.1)
Education level	No formal education	27 (4.0)
	Primary	185 (27.1)
	Secondary	385 (56.5)
	Tertiary	85 (12.5)
Monthly income	≤24,900 NGN (~$2/day)	176 (25.8)
	>24,900 NGN	506 (74.2)
Family history of HTN	Yes	641 (94.0)
	No	41 (6.0)
Hypertension	Hypertensive	227 (33.3)
	Non-hypertensive	455 (66.7)

Table 2 shows the distribution of anthropometric features among study participants. The individuals' obesity was measured using three indicators: BMI, WC, and WHR, all of which were based on the WHO's measuring standard scale. Using the BMI, less than half of the participants were obese (324, 47.5%). Based on WC, obesity was observed in 71 (51.1%) of women compared to 43 (7.9%) of men. Similarly, according to the WHR, 131 (94.2%) of women and 326 (60%) of men were classified as obese.

**Table 2:** Frequency distribution of body fat distribution of study participants (N = 682).

Variable	Category	*n* (%)
Body mass index (BMI)	Normal (<25)	358 (52.5)
	Obese (≥25)	324 (47.5)
Waist circumference (Male)	Normal (<102 cm)	500 (92.1)
	Obese (≥102 cm)	43 (7.9)
Waist circumference (Female)	Normal (<88 cm)	68 (48.9)
	Obese (≥88 cm)	71 (51.1)
Waist-to-hip ratio (Male)	Normal (<0.90)	217 (40.0)
	Obese (≥0.90)	326 (60.0)
Waist-to-hip ratio (Female)	Normal (<0.80)	8 (5.8)
	Obese (≥0.80)	131 (94.2)

Table 3 shows the frequency and percentage distribution of behavioral/lifestyle characteristics of study participants. A total of 11 (16.9%) were smokers, 283 (41.5%) consumed alcohol, and 377 (55.3%) reported poor sleep quality. Regarding physical activity, 222 (32.6%) were physically inactive, while 460 (67.4%) engaged in at least 600 MET-minutes of activity per week. These findings highlight the high prevalence of modifiable lifestyle risk factors for hypertension among transport workers.

**Table 3:** Frequency distribution of behavioral/lifestyle characteristics of study participants (N = 682).

Variable	Category	*n* (%)
Smoking status	Yes	115 (16.9)
	No	567 (83.1)
Alcohol use	Yes	283 (41.5)
	No	399 (58.5)
Physical activity	Inactive	222 (32.6)
	Active	460 (67.4)
Sleep quality	Poor	377 (55.3)
	Good	305 (44.7)

Table 4 presents the relationship between participants’ sociodemographic factors and the occurrence of hypertension. The prevalence of hypertension was found to be highest within the older age categories (16, 45.7%) in the ≥65 years age group, followed by 123 (45.2%) in the 45-64 years age group, while the lowest prevalence (18, 14.9%) was recorded in the <25 years age group. The difference was statistically significant. The prevalence of hypertension was higher among married respondents (182, 33.5%) compared to unmarried respondents (29, 19%). The difference is significant. The nature of work of the respondents was statistically significant, with prevalence higher among transporters (166, 35.5%) compared to non-transporters (61, 28.4%). Respondent's level of education was statistically significant with hypertension (*P* < 0.001). Respondents with no formal education had the highest prevalence (16, 59.3%), followed by respondents with primary education (69, 37.3%), while respondents with tertiary education (22, 25.9%) had the least prevalence of hypertension. Also, average income was statistically associated with hypertension (*P *< 0.05).

**Table 4:** Distribution of hypertension status by sociodemographic characteristics of study participants (N = 682). *P-value < 0.01. **P-value < 0.05. HTN, hypertension; NG, Nigerian Naira

Variable	Category	Hypertensive, *n* (%)	Not hypertensive, *n* (%)	*χ*²-value	*P*-value
Age (years)	<25	12 (17.1)	58 (82.9)	46.822	<0.001*
	24-34	18 (14.9)	103 (85.1)		
	35-44	58 (31.5)	126 (68.5)		
	45-64	123 (45.2)	149 (54.8)		
	≥65	16 (45.7)	19 (54.3)		
Gender	Male	182 (33.5)	361 (66.5)	0.065	0.841
	Female	45 (32.4)	94 (64.6)		
Marital status	Unmarried	29 (19.0)	124 (81.0)	18.241	<0.001*
	Married	198 (37.4)	331 (62.6)		
Occupation	Transporter	166 (35.5)	301 (64.5)	3.412	0.038**
	Non-transporter	61 (28.4)	154 (71.6)		
Ethnicity	Yoruba	222 (33.6)	439 (66.4)	0.876	0.482
	Others	5 (23.8)	16 (76.2)		
Education level	No formal education	16 (59.3)	11 (40.7)	12.418	0.006*
	Primary	69 (37.3)	116 (62.7)		
	Secondary	120 (31.2)	265 (68.8)		
	Tertiary	22 (25.9)	63 (74.1)		
Monthly income	≤24,900 NGN (~$2/day)	45 (25.6)	131 (74.4)	6.361	0.012**
	>24,900 NGN	182 (36.0)	324 (64.0)		
Family history of HTN	Yes	215 (33.5)	426 (66.5)	0.317	0.574
	No	12 (29.3)	29 (70.7)		

The BMI of the respondents was statistically associated with the prevalence of hypertension (*P *< 0.001) among the transport workers, with those having an abnormal BMI showing a higher prevalence (129, 39.8%) compared to those with a normal BMI (98, 27.4%). Also, WC and WHR were statistically associated with the prevalence of hypertension, with a higher prevalence observed among obese respondents (47, 41.2%, and 92, 41.6%) compared to those with normal WC and WHR (180, 31.7%, and 135, 29.3%), respectively (Table 5).

**Table 5:** Distribution of hypertension status by body fat distribution of study participants (N = 682). *P-value < 0.01. **P-value < 0.05.

Variable	Category	Hypertensive, *n* (%)	Not hypertensive, *n* (%)	*χ*²-value	*P*-value
Body mass index (BMI)	Normal (<25)	98 (27.4)	260 (72.6)	11.854	0.001*
	Obese (≥25)	129 (39.8)	195 (60.2)		
Waist circumference	Normal	180 (31.7)	388 (68.3)	3.472	0.049**
	Obese	47 (41.2)	67 (58.8)		
Waist-to-hip ratio (WHR)	Normal	135 (29.3)	326 (70.7)	10.252	0.001*
	Obese	92 (41.6)	129 (58.4)		

The prevalence of hypertension was higher among smokers (43, 37.4%) compared to non-smokers at 184 (32.5%). The difference was, however, not statistically significant (*P *= 0.305). Respondents with a history of smoking (72, 41.1%) had a higher prevalence of hypertension than respondents with no history of smoking, with the difference statistically significant (*P *= 0.001). Respondents who consumed alcohol had a higher prevalence of hypertension (115, 40.5%) compared to those who did not consume alcohol (112, 28.1%). The difference was found to be statistically significant (*P *= 0.001) (Table 6).

**Table 6:** Distribution of hypertension status using participants' lifestyle characteristics.

Variable	Category	Hypertensive, *n* (%)	Not hypertensive, *n* (%)	*χ*²-value	*P*-value
Smoking status	Yes	43 (37.4)	72 (62.6)	1.051	0.305
	No	184 (32.5)	383 (67.5)		
History of smoking	Yes	72 (41.1)	103 (58.9)	6.547	0.011*
	No	155 (30.6)	352 (69.4)		
Alcohol consumption	Yes	115 (40.5)	168 (59.4)	11.773	0.001**
	No	112 (28.1)	287 (71.9)		
Sleep quality	Poor	93 (30.5)	212 (69.5)	1.938	0.164
	Good	134 (35.5)	243 (64.5)		
Physical inactivity	Yes	69 (31.1)	153 (68.9)	0.72	0.396
	No	158 (34.3)	302 (65.7)		

**Figure 2:**
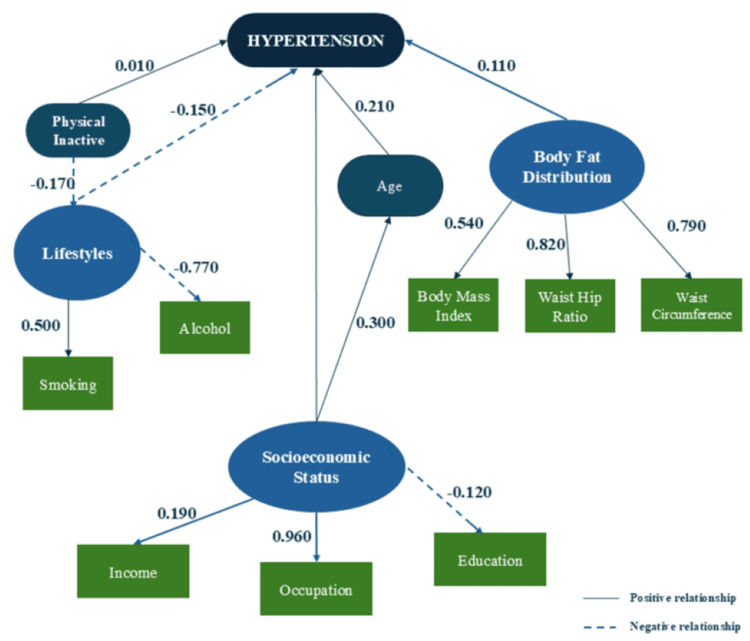
Structural Equation Model path diagram showing standardized coefficients among socioeconomic, lifestyle, and body fat distribution constructs associated with hypertension among transport workers in Ibadan, Nigeria. Solid arrows represent positive relationships, while dashed arrows represent negative relationships. Image credit: Created by Olajide Akinpeloye using data analyzed in AMOS (SPSS) and visually refined in Microsoft PowerPoint for clarity.

The structural equation model path diagram (Figure 2) illustrates the standardized path coefficients among the latent variables and their observed indicators. The model produced an admissible solution; therefore, standardized error terms were reported for each indicator variable, confirming that the model provides a good fit for the dataset. The standardized coefficients of the sociodemographic (β = 0.01) and body fat distribution (β = 0.11) constructs were positively related to hypertension, while the lifestyle construct was negatively related (β = −0.15). The model demonstrated a satisfactory fit to the data, as reflected by the fit indices: χ²/df = 4.704, RMSEA = 0.04, GFI = 0.898, CFI = 0.911, and TLI = 0.896.

The SEM results in Table 7 indicate that age (β = 0.21, *P* = 0.003) was the strongest direct predictor of hypertension, followed by body fat distribution (β = 0.11, *P* = 0.030) and lifestyle (β = −0.15, *P* = 0.007). Among lifestyle factors, alcohol (β = −0.77, *P* = 0.004) and physical inactivity (β = −0.17, *P* = 0.012) were significant, while smoking contributed positively. And sleep quality was not significant. Central obesity indicators (WHR and WC) exhibited stronger effects than BMI. Socioeconomic indicators such as occupation, sex, education, and income also contributed, though their direct effect on hypertension was small. Overall, hypertension risk among transport workers was shaped primarily by age, central obesity, and lifestyle factors, with socioeconomic status exerting both direct and indirect influences through these pathways.

**Table 7:** Structural Equation Model showing standardized path coefficients, standard errors, and 95% confidence intervals for factors associated with hypertension among transport workers in Ibadan, Nigeria. CIs are presented only for key direct predictors of hypertension to reflect estimation precision. Positive coefficients denote direct relationships, while negative coefficients indicate inverse relationships between constructs. β, standardized path coefficient; SE, standard error; CI, 95% confidence interval

Indicator	Latent variable	Estimate (β)	SE	*P*-value	95% CI
Physical inactivity	Lifestyle	−0.170	0.026	0.012	-
Smoking	Lifestyle	0.500	-	-	-
Alcohol	Lifestyle	−0.770	0.739	0.004	-
Sleep	Lifestyle	−0.024	0.136	0.623	-
Body mass index	Body fat distribution	0.540	-	-	-
Waist-to-hip ratio	Body fat distribution	0.820	0.076	<0.001	-
Waist circumference	Body fat distribution	0.790	0.058	<0.001	-
Age	Socioeconomic status	0.300	-	0.004	-
Sex	Socioeconomic status	−0.670	-	0.002	-
Occupation	Socioeconomic status	0.960	-	0.002	-
Education	Socioeconomic status	−0.120	-	0.003	-
Income	Socioeconomic status	0.190	-	0.032	-
Family history	Socioeconomic status	0.120	-	-	-
Hypertension	Socioeconomic status	0.010	0.658	0.01	(0.002, 0.018)
Hypertension	Body fat distribution	0.110	0.048	0.03	(0.011, 0.209)
Hypertension	Lifestyle	−0.150	0.133	0.007	(−0.259, −0.041)
Hypertension	Age	0.210	0.017	0.003	(0.071, 0.349)
Hypertension	Physical inactivity	0.010	0.038	<0.001	(0.004, 0.016)

Table 8 summarizes the fit indices for the model. The goodness-of-fit test was significant (*P* < 0.05). The relative chi-square, which is highly influenced by sample size, was less than 5 (χ²/df = 4.90; RMSEA = 0.03; GFI = 0.91; CFI = 0.90; TLI = 0.90). The information criterion (AIC = 667.929) also indicates that the model provides a good fit to the dataset.

**Table 8:** Confirmatory Factor Analysis (CFA) summary table of model fit indices. RMSEA, root mean square error of approximation; GFI, Goodness-of-Fit Index; TLI, Tucker-Lewis Index; CFI, Comparative Fit Index; AIC, Akaike Information Criterion

Fit indices	Model estimate
χ^2 ^(df)	357.7 (73)
*P*-value	<0.000
RMSEA	0.03
GFI	0.91
TLI	0.90
CFI	0.90
AIC	667.929

Table 9 reveals that age exerted the strongest overall influence on hypertension (β = 0.214), followed by body fat distribution (β = 0.114), lifestyle factors (β = −0.147), sociodemographic attributes (β = 0.069), and physical inactivity (β = 0.032) when both direct and indirect pathways were considered. The negative path coefficient for lifestyle (β = −0.15) indicates that healthier lifestyle behaviors, such as reduced alcohol consumption, increased physical activity, and lower tobacco use, were associated with a lower likelihood of hypertension. This inverse relationship aligns with previous findings that lifestyle modification mitigates cardiovascular risk. However, the negative coefficient may also reflect the coding direction used in the model, where higher scores on the lifestyle construct represented less favorable behaviors. Moreover, age and socioeconomic status may mediate this pathway, as older or lower-income individuals are more likely to report unhealthy lifestyle habits, thereby indirectly contributing to increased hypertension risk.

**Table 9:** Direct, indirect, and total effects of variables on hypertension

Variable	Direct	Indirect	Total
Bodyfat	0.114	No path	0.114
Physical	0.006	0.026	0.032
SES	0.006	0.063	0.069
Age	0.214	No path	0.214
Lifestyle	-0.147	No path	-0.147

## Discussion

The respondents were aged 18-67 years, with most between 45 and 64 years, followed by those aged 35-44 years. The mean age of 42.4 years closely matches the 45.3 years reported among commercial drivers in Ibadan [4]. Inter-state drivers formed 467 (68.5%) of participants, a group considered at high risk of hypertension where fitness for duty is crucial [9]. Over half (385, 56.5%) had secondary education, consistent with findings that secondary schooling is most common among transport workers and can influence job performance [29].

Given the economic importance of commercial drivers, assessing hypertension and its risk factors is critical [9]. The hypertension prevalence of 227 (33.3%) in this study exceeds the global estimate of 31.1 % and Africa’s 30.8% and is higher than Nigeria’s pooled crude prevalence of 30.6% [30]. It also surpasses the 9.0% found among commercial drivers in Abuja [9] and the 27.7% reported in south-western Nigeria [4], while remaining below the 40.0% documented among truck drivers in South India [31]. The figure still falls within the broad Nigerian national range of 8%-46.4% described in earlier reviews [32].

Sociodemographic characteristics were strongly associated with hypertension. Older age conferred a higher risk, and married participants (198, 37.4%) were more hypertensive than unmarried participants (29, 19.0%). Occupation also mattered: commercial drivers, conductors, and park managers (166, 35.5%) had a higher prevalence of hypertension compared to nearby traders (61, 28.4%). These findings confirm that commercial drivers are a high-risk group [9]. Education and income also played a role: participants with no formal education (16, 59.3%) and those with higher income (182, 36.0%) were more likely to have hypertension, echoing evidence that limited education and low socioeconomic status increase hypertension risk [12].

Body fat distribution showed a significant positive relationship with hypertension. High BMI, WC, and WHR all increased risk, with 129 (39.8%) of hypertensive transport workers having a BMI above 25 kg/m² compared to 98 (27.4%) with normal BMI. These results align with research linking increasing BMI and age to hypertension and identify WHR as a predisposing factor regardless of sex or region [33].

Lifestyle and behavioral factors were equally important. Respondents with a history of smoking (72, 41.1%) had a higher prevalence of hypertension compared to those with no smoking history (155, 30.6%). Similarly, participants who consumed alcohol (115, 40.5%) were more hypertensive than non-drinkers (112, 28.1%). Poor sleep quality was also common, with 93 (30.5%) of those with poor sleep being hypertensive compared to 134 (35.5%) of those reporting good sleep. These findings underscore the need for healthy lifestyle changes to prevent and manage hypertension.

SEM confirmed that sociodemographic, metabolic, and lifestyle constructs each had significant direct effects on hypertension. Sociodemographic status also exerted indirect effects via lifestyle and metabolic factors, indicating that obesity, physical inactivity, and heavy alcohol use can mediate the influence of age, income, family history, and education. The hypothesized structural model fit the data well, with all variables significantly related to their respective constructs at the 5% level, supporting similar conclusions in related modeling studies [34].

This study has several limitations. Being cross-sectional in design, it cannot establish causal relationships between the identified risk factors and hypertension. Information on lifestyle behaviors such as smoking, alcohol use, and sleep quality was self-reported, which may introduce recall or social desirability bias. Furthermore, the sample was limited to transport workers in Ibadan, which may restrict the generalizability of the findings to other populations or regions. Notably, females accounted for only about 20% of participants, reflecting the male-dominated nature of the transport sector. Future studies should explore gender-specific risk factors and behaviors, as female transport workers may exhibit different exposure patterns and physiological responses to hypertension risk factors. Despite these limitations, the relatively large sample size and the application of SEM strengthen the internal validity of the results and provide valuable insights into the multidimensional determinants of hypertension.

From a policy perspective, these findings underscore the need for workplace-based interventions tailored to transport workers. Regular blood pressure screening, targeted health education on diet and physical activity, and stress management initiatives could substantially reduce hypertension risk in this occupational group. Integrating such preventive strategies into transport unions or company health programs may improve cardiovascular outcomes and contribute to achieving broader public health goals related to non-communicable disease prevention.

## Conclusions

Hypertension was highly prevalent among transport workers in Ibadan and was influenced by sociodemographic, lifestyle, and body fat distribution factors. Age and central obesity were the strongest predictors, while modifiable lifestyle behaviors such as smoking, alcohol intake, and physical inactivity also played important roles. These findings underscore the need for workplace health programs and targeted interventions to promote healthy lifestyles and reduce hypertension risk in this occupational group. Practical implementation could include integrating routine blood pressure screening into driver licensing procedures and transport union welfare programs, along with continuous education on diet, exercise, and stress management.
